# The chromosome 9p21 variant interacts with vegetable and wine intake to influence the risk of cardiovascular disease: a population based cohort study

**DOI:** 10.1186/s12881-014-0138-x

**Published:** 2014-12-31

**Authors:** George Hindy, Ulrika Ericson, Viktor Hamrefors, Isabel Drake, Elisabet Wirfält, Olle Melander, Marju Orho-Melander

**Affiliations:** Diabetes and Cardiovascular Disease-Genetic Epidemiology, Lund, Sweden; Hypertension and Cardiovascular Disease, Lund, Sweden; Nutrition Epidemiology, Department of Clinical Sciences in Malmö, Lund University, Lund, Sweden

**Keywords:** Cardiovascular disease, Chromosome 9p21, Diet, Gene, Gene–diet interactions

## Abstract

**Background:**

Chromosome 9p21 variants are associated with cardiovascular disease (CVD) but not with any of its known risk markers. However, recent studies have suggested that the risk associated with 9p21 variation is modified by a prudent dietary pattern and smoking. We tested if the increased risk of CVD by the 9p21 single nucleotide polymorphism rs4977574 is modified by intakes of vegetables, fruits, alcohol, or wine, and if rs4977574 interacts with environmental factors on known CVD risk markers.

**Methods:**

Multivariable Cox regression analyses were performed in 23,949 individuals from the population-based prospective Malmö Diet and Cancer Study (MDCS), of whom 3,164 developed CVD during 15 years of follow-up. The rs4977574 variant (major allele: A; minor allele: G) was genotyped using TaqMan® Assay Design probes. Dietary data were collected at baseline using a modified diet history method. Cross-sectional analyses were performed in 4,828 MDCS participants with fasting blood levels of circulating risk factors measured at baseline.

**Results:**

Each rs4977574 G allele was associated with a 16% increased incidence of CVD (95% confidence interval (CI), 1.10–1.22). Higher vegetable intake (hazard ratio (HR), 0.95 [CI: 0.91–0.996]), wine intake (HR, 0.91 [CI: 0.86–0.96]), and total alcohol consumption (HR, 0.92 [CI: 0.86–0.98]) were associated with lower CVD incidence. The increased CVD incidence by the G allele was restricted to individuals with medium or high vegetable intake (P_interaction_ = 0.043), and to non- and low consumers of wine (P_interaction_ = 0.029). Although rs4977574 did not associate with any known risk markers, stratification by vegetable intake and smoking suggested an interaction with rs4977574 on glycated hemoglobin and high-density lipoprotein cholesterol (P_interaction_ = 0.015 and 0.049, respectively).

**Conclusions:**

Our results indicate that rs4977574 interacts with vegetable and wine intake to affect the incidence of CVD, and suggest that an interaction may exist between environmental risk factors and rs4977574 on known risk markers of CVD.

**Electronic supplementary material:**

The online version of this article (doi:10.1186/s12881-014-0138-x) contains supplementary material, which is available to authorized users.

## Background

The single nucleotide polymorphisms (SNPs) on chromosome 9p21 identified by genome-wide association studies are the strongest known to date to be associated with coronary artery disease (CAD) [1-3] and myocardial infarction (MI) [4,5]. Variants at this locus have also been associated with abdominal aortic aneurysms, intracranial aneurysms [6], and periodontitis [7]. Approximately 25% of the Caucasian population is homozygous for an allele at this locus that confers a 30–40% increased risk of developing CAD [2]. However, because this risk increase is independent of all conventional risk factors such as plasma lipoprotein levels, diabetes, hypertension, or markers of inflammation, the mechanism by which the 9p21 locus confers risk remains poorly understood [2,4].

Several dietary factors have been shown to associate with cardiovascular disease (CVD). For instance, a dietary intake of vegetables and nuts, and dietary patterns such as the Mediterranean-like diet are associated with protection against CVD, while an intake of *trans*-fatty acids, a high glycemic load and glycemic index diets have shown harmful effects [8]. Previous observations from the Malmö Diet and Cancer Study (MDCS) found that a high quality diet (i.e., in line with current dietary recommendations) is associated with lower CVD risk [9]. Strong evidence from the Nurses’ Health study and the Health Professionals’ Follow-up Study suggests that diets rich in vegetables and fruits lower the risk of CVD [10]. Moreover, two large meta-analyses combining prospective studies from the United States and Europe confirmed the protective association of higher fruit and vegetable intake with incident CAD and stroke [11,12]. Additionally, moderate alcohol and wine consumption has been recognized as protective against CVD. Moderate alcohol intake has been associated with a lower risk of multiple cardiovascular outcomes in a systemic review and meta-analysis of 84 studies, while high alcohol consumption was associated with increased cardiovascular mortality and stroke incidence, consistent with U- or J-shaped curves [13]. Compelling evidence also exists for wine consumption providing a more pronounced cardio-protective effect compared with other alcoholic beverages [14,15].

A recent multi-ethnic study reported that the risk of MI and CVD determined by 9p21 variants was modified by a prudent diet score mainly driven by the raw vegetable component [16]. Another MDCS finding was of the reported interaction between 9p21 rs4977574 variant and smoking on the CAD incidence: a strong association was only observed among nonsmokers, while this association was significantly attenuated among smokers [17]. In the present study, we tested if the associated effect of the chromosome 9p21 rs4977574 variant on the risk of CVD was modified by vegetable, fruit, wine, or total alcohol intake. We further investigated whether these environmental factors interacted with the 9p21 variant on known risk markers of CVD.

## Methods

### Study population

#### Prospective study cohort

The MDCS is a population-based prospective cohort in the city of Malmö, Sweden. Individuals born between 1923 and 1950 were recruited from a source population of 74,138 residing in Malmö with Swedish reading and writing skills. The baseline examination period extended between March 1991 and October 1996. Written informed consent was obtained from all participants on their first visit to the hospital and after receiving instructions about the study procedures and questionnaires. Anthropometrics, body composition, and blood pressure were directly measured. Study participants were instructed on how to fill in a diet questionnaire, a diet diary, and an extensive questionnaire covering socioeconomic and lifestyle factors including medical history, smoking habits, education, and physical activity. Participants returned approximately 2 weeks later for a diet history interview. By the end of the baseline examination period, complete dietary, anthropometric, and lifestyle data had been collected on 28,098 participants. Details of the recruitment procedures are described elsewhere [18].

We excluded individuals with prevalent CVD and diabetes. Prevalent CVD cases were identified as individuals with a history of MI or stroke. Prevalent diabetes cases were identified as individuals with a self-reported diabetes diagnosis or those on a self-reported anti-diabetic regimen. Our study population included 23,949 individuals free from CVD or diabetes with available DNA samples and successfully genotyped for the rs4977574 SNP at the chromosome 9p21 locus.

#### Cross-sectional study

At baseline, 6,103 individuals were randomly selected from the whole MDCS (N = 28,098) to participate in the Malmö Diet and Cancer Cardiovascular Cohort (MDC-CC). These individuals underwent additional baseline measurements from fasting blood samples that included low-density lipoprotein cholesterol (LDLC), high-density lipoprotein cholesterol (HDLC), triglycerides (TG), fasting blood glucose, and high sensitivity C-reactive protein (hsCRP). From this population we excluded individuals with prevalent diabetes, and prevalent CVD (identified as before). Our cross-sectional study included 4,828 individuals with complete data on rs4977574 genotype, diet, fasting blood tests, and blood pressure. The MDCS was approved by the Ethical Committee at Lund University (LU 51–90). All participants provided written informed consent.

### Incident cardiovascular disease assessment

Incident CVD cases were identified as individuals with CAD (defined as fatal or nonfatal MI or death due to ischemic heart disease) or individuals with fatal or non-fatal stroke using three registers: the Swedish Hospital Discharge Register, the Swedish Cause of Death Register, and the Stroke Register of Malmö. The identification of these register end-points has been described and validated elsewhere [19-21]. The follow-up period for the present study extended to December 31, 2010. MI cases were defined using codes 410 and I21 of the 9^th^ and 10^th^ revisions of the International Classification of Diseases (ICD9 and ICD10), and the stroke cases were defined using codes 430, 431, 434, and 436 of the ICD9, and codes I60, I61, I63, and I64 of the ICD10. A total of 3,164 individuals developed CVD, 1,844 developed CAD, and 1,556 developed stroke over a mean follow-up period of 15 years.

### Clinical measurements

A balance-beam scale was used to measure weight (in kg) with subjects wearing light clothing and no shoes. A fixed stadiometer was used to measure height (in cm). Body mass index (BMI) was measured as weight (kg) divided by height (m^2^). Waist circumference (in cm) was measured midway between the lowest rib margin and iliac crest. Fasting serum lipids, whole blood glucose, glycated hemoglobin (HbA_1C_), and inflammatory markers were measured from blood samples drawn after an overnight fast. Fasting blood glucose was converted to fasting plasma glucose (FPG) by multiplying the values by 1.13. HbA_1C_ values were converted from the Swedish Mono-S standardization system to the International Federation of Clinical Chemistry and Laboratory Medicine (IFCC) units using the following formula: IFCC = (10.11 × Mono-S) − 8.94. Samples were analyzed by routine standard methods at the Department of Clinical Chemistry, Malmö University Hospital. Fasting blood measurements were only available in MDC-CC.

### Dietary assessment

The MDCS had a specially designed modified diet history method that consisted of a 7-day menu book, a 168-item questionnaire, and a 45–60 min diet history interview. All cooked meals, cold beverages including alcohol, medicinal drugs, natural remedies, and nutrient supplements consumed were recorded in the menu book. Participants recorded meal patterns, consumption frequencies, and portion sizes of regularly consumed foods not covered by the menu book in the questionnaire. A 48-page booklet was used to help participants estimate portion sizes. Diet history interviews were performed by trained interviewers, during which portion sizes and dishes were estimated using a more extensive book containing photographs. Participants were also asked about their meal patterns, cooking methods, and food choices.

The average daily intake of foods was calculated using data from the menu book and diet questionnaire, then converted into energy and nutrient intakes using the MDC Food and Nutrient Database. This was designed for the MDCS and was derived from the PC KOST2-93 database of the Swedish National Food Administration [22,23].

In September 1994, the coding routines for dietary data were modified slightly to shorten the interview time (from 60 to 45 minutes). This resulted in two slightly different method versions (before and after September 1994) without any major influence on the ranking of individuals [23]. A method variable, classifying data collected before and after September 1994, and a four-category season variable (i.e. winter, spring, summer, and autumn) were created and used as covariates to adjust for variation in data collection over time. The relative validity of the dietary assessment method in MDCS was previously evaluated in a sample of 206 (105 women and 101 men) Malmö residents, aged 50–69 years. The reference method was based on weighed food records taken for 3 days every second month over the course of 1 year. Crude Pearson correlation coefficients for vegetable, fruit, wine, and total alcohol intake were 0.68, 0.60, 0.50, and 0.78, respectively, for men, and 0.58, 0.77, 0.65, and 0.83, respectively, for women [24,25].

The dietary exposure variables used were vegetables, fruit, wine, and total alcohol intake. Vegetable intake included all raw, dried, and cooked vegetables. Fruit intake included all citrus and non-citrus fruits and berries, in addition to dried fruits. The weights of dried fruits were corrected for their lower water content. Fruit and vegetable intakes were estimated as g per day and ranked into tertiles. The wine variable included red, white, and fortified wines. Non-consumers of wine were defined as those reporting no consumption of wine in the menu book, and indicating no consumption of wine during the previous month in the questionnaire. Wine non-consumers were categorized into a separate group and the rest were stratified into two groups using the median split. Total alcohol intake was classified into the following four categories based on g of alcohol consumed per day: abstainers (those reporting zero consumption of alcohol in the menu book, and indicating no consumption of alcohol in the previous year in the questionnaire), low (<15 g/d in women or <20 g/d in men), medium (15–30 g/d in women or 20–40 g/d in men), and high (>30 g/d in women or >40 g/d in men). This categorization was based on an assumption of biological risk [26]. For sensitivity analysis, we excluded abstainers and created tertiles of total alcohol consumption.

### Other variables used as potential confounders

Leisure time physical activity was assessed by an extensive lifestyle questionnaire adapted from the Minnesota Leisure Time Physical Activity Questionnaire. Participants estimated the amount of time (in min) they spent performing each of 17 different physical activities per week for each season. The duration was multiplied by an intensity factor to create a physical activity score that was divided into tertiles. Participants were also classified as current smokers, former smokers, and never-smokers. The education variable was created by classifying participants according to their highest educational level (≤8 years, 9–10 years, and 11–13 years at school, and university degree).

### Genotyping

The chromosome 9p21 rs4977574 SNP (major allele: A; minor allele: G) was genotyped using TaqMan® Assay Design probes with a real-time PCR assay using ABI-7900HT equipment (Applied Biosystems, Foster City, CA) according to the manufacturer’s instructions. The concordance rate was more than 99.9% in randomly selected samples (20%) and the genotypes were in Hardy–Weinberg equilibrium (*P* = 0.18). We selected the rs4977574 for our study as this SNP provided the strongest evidence for association with early-onset MI in a large genome-wide association study (GWAS) by Kathiresan et al. [5] and as the same SNP has earlier been used in two other interaction studies [16,17]. This SNP is in high linkage disequilibrium (r^2^ = 0.91–0.97) with the four SNPs (rs10757278, rs1333049, rs10757274, and rs2383206) originally identified in the discovery GWAS studies [1,2,4].

### Statistical analysis

PASW statistics version 21 software (SPSS Inc., Chicago, IL) was used for statistical analyses. The prospective additive genetic association of the chromosome 9p21 rs4977574 G allele with incident CVD was assessed using a multivariable Cox proportional hazards model adjusting for age and sex. In cross-sectional analyses, cardio-metabolic traits and dietary intakes (dependent variables) were examined according to rs4977574 genotype (independent variable) using linear regression analyses with adjustments for age and sex. Prospective associations between tertiles of vegetable and fruit intakes, categories of alcohol or wine intake, and incidence of CVD were assessed in a multivariable Cox proportional hazards model with adjustments for age, sex, total energy intake, season, dietary assessment method, BMI, systolic blood pressure (SBP), use of lipid-lowering medication, use of anti-hypertensive medication, tertiles of leisure time physical activity, smoking status, level of education, and total alcohol intake categories when applicable. In cross-sectional analyses, cardio-metabolic traits and dietary intakes (dependent variables) were examined according to intake categories of vegetables, fruits, wine and alcohol (independent variables), using a multivariable linear regression model with the same adjustments as listed above.

Interactions between rs4977574 and tertiles of vegetable and fruit intakes, and the categories of alcohol or wine intake on incidence of CVD were tested by including both the rs4977574 variable and the environmental variables and their multiplicative factor in the multivariable models, using the same adjustments described above. The tertiles of vegetable and fruit intakes and the categories of wine and total alcohol intakes were treated as continuous variables. Significant prospective interactions between environmental factors and rs4977574 on the incidence of CVD were further explored by cross-sectional interaction analyses between rs4977574 and quantitative glycemic, lipid, and inflammatory traits measured at baseline with the same adjustments described above.

Because it may be argued that BMI, SBP, and the use of lipid-lowering or anti-hypertensive medication could be on the causal pathway and should not be included in the adjustment models, we additionally performed all of the above analyses excluding these variables as possible confounders. Additional analyses were performed by mutually adjusting the analyses for correlated environmental factors such as vegetable and wine intake, and wine consumption and total alcohol consumption. The *P* values of the study were not corrected for multiple comparisons; those < 0.05 were considered nominally statistically significant. All reported *P* values are two-sided.

## Results

The rs4977574 G allele was associated with an increased incidence of CVD (hazard ratio [HR]: 1.16 per G allele; 95% confidence interval [CI]: 1.10–1.22) in MDCS (Table 1), while higher vegetable, wine, and total alcohol intakes were associated with a decreased incidence of CVD (HR, 0.95 [CI: 0.91–0.996], 0.91 [0.86–0.96], and 0.92 [0.86–0.98], respectively). Cross-sectional associations of intakes of vegetables, fruits, wine and alcohol with baseline levels of different cardio-metabolic biomarkers are shown in Additional file 1: Table S1–S4.

**Table 1:** **Characteristics of the Malmö Diet and Cancer Study population by rs4977574 genotype**

	**rs4977574 genotype**			**HR (95% CI)** ^**a**^	**P**_**trend**_
	**AA**	**AG**	**GG**	**or β (SE)** ^**b**^	
Total number	7325	11777	4847		
Sex (%women)	62.6	62.3	62.3		
Incident CVD *N* (%)	863 (11.8)	1562 (13.3)	739 (15.3)	1.16 (1.10-1.22)	4 × 10^−09^
Age (years)	57.9 ± 7.7	57.9 ± 7.6	57.8 ± 7.7	−0.04 (0.07)	0.60
BMI (kg/m^2^)	25.7 ± 3.9	25.6 ± 3.9	25.7 ± 3.9	0.001 (0.035)	0.98
Waist (cm)	83.6 ± 15.6	83.4 ± 12.8	83.7 ± 16.7	0.02 (0.11)	0.87
SBP (mmHg)	141 ± 20	141 ± 20	141 ± 20	0.21 (0.17)	0.21
DBP (mmHg)	85 ± 10	85 ± 10	86 ± 10	0.03 (0.09)	0.76
FPG (mmol/L)^c^	5.63 ± 0.84	5.62 ± 0.72	5.66 ± 0.91	0.009 (0.016)	0.59
HbA_1C_ (mmol/mol)^c^	39.6 ± 4.94	39.6 ± 4.82	39.9 ± 5.32	0.13 (0.10)	0.19
LDLC (mmol/L)^c^	4.15 ± 1.00	4.19 ± 0.98	4.19 ± 0.97	0.02 (0.02)	0.33
HDLC (mmol/L)^c^	1.39 ± 0.37	1.40 ± 0.38	1.39 ± 0.36	0.001 (0.007)	0.85
Triglycerides (mmol/L)^c^	1.35 ± 0.73	1.32 ± 0.71	1.37 ± 0.90	0.002 (0.015)	0.48
hsCRP^c^	0.26 ± 0.43	0.24 ± 0.39	0.27 ± 0.48	0.003 (0.009)	0.67
Total energy intake (kcal/day)	2280 ± 652	2274 ± 653	2276 ± 653	−3.90 (5.26)	0.46
Vegetables (g/day)	180 ± 98	182 ± 100	180 ± 99	−0.18 (0.90)	0.85
Fruits (g/day)	196 ± 127	193 ± 125	197 ± 127	0.29 (1.14)	0.80
Wine (g/day)	41.2 ± 58.2	42.8 ± 70.0	42.5 ± 60.6	0.70 (0.55)	0.20
Alcohol (g/day)	10.6 ± 12.3	10.9 ± 12.7	10.8 ± 12.8	0.13 (0.11)	0.22

Data represented as mean ± standard deviation.

^a^Multivariable Cox proportional hazards model for incidence of cardiovascular disease (CVD) during follow-up adjusting for age and sex with HR referring to hazard ratio per risk G allele using an additive genetic model.

^b^Linear regression analyses of rs4977574 G allele using an additive genetic model with quantitative traits or characteristics at baseline adjusting for age and sex when appropriate with β referring to associated effect estimate per risk G allele.

^c^In MDC-CC only, N = 4,828 (AA N = 1,460; AG N = 2,381; GG N = 987).

CI, Confidence Interval; CVD, cardiovascular disease; SE, standard error; BMI, Body Mass Index; SBP, Systolic Blood Pressure; DBP, Diastolic Blood Pressure; FPG, Fasting Plasma Glucose; HbA_1C_, Hemoglobin A_1C_; LDLC, Low-Density Lipoprotein Cholesterol; HDLC, High-Density Lipoprotein Cholesterol; hsCRP, High-Sensitivity C-Reactive Protein.

The CVD risk increase by the rs4977574 G allele was modified by vegetable and wine intakes (P_interaction_ = 0.043 and 0.029, respectively) but not with fruit or total alcohol intakes (P_interaction_ = 0.69 and 0.73, respectively). The association between the rs4977574 G allele and increased incidence of CVD was restricted to the medium and high tertiles of vegetable intake (P_trend_ = 6 × 10^−8^ and 0.0004, respectively) and to non- and low consumers of wine (P_trend_ = 4 × 10^−7^ and 0.0004, respectively). The inverse association between higher vegetable intake and CVD incidence was restricted to individuals with no risk alleles (AA genotype, P_trend_ = 0.001), while the inverse association between higher wine consumption and incidence of CVD was restricted to carriers of rs4977574 G alleles (P_trend_ = 0.00009) (AG and GG genotypes, P_trend_ = 0.01 and 0.001, respectively) (Table 2). Both vegetable and wine intake modified the risk of both CAD and stroke by rs4977574 variant in a similar fashion as observed with total CVD. However, the tests of interaction did not reach statistical significance (Additional file 1: Table S6 and S7).

**Table 2:** **Hazard ratio for incident CVD in the Malmö Diet and Cancer Study (n = 23,949) according to rs4977574 genotype and dietary or alcohol intake categories**

		**Hazard ratio (95% Confidence interval)**				**P**_**trend**_^**b,d**^	**P**_**interaction**_^**e,f**^
		**AA**	**AG**	**GG**	**Additive model** ^**b,d**^		
	*n*	7325	11777	4847			
**Vegetables (g/day)**							0.043, 0.049
Low	7952	1.29 (1.09–1.52)	1.22 (1.04–1.43)	1.50 (1.26–1.79)	1.06 (0.98–1.14)	0.16	
Medium	8041	0.87 (0.73–1.05)	1.27 (1.09–1.48)	1.41 (1.18–1.70)	1.27 (1.17–1.38)	5.6 × 10^−8^	
High	7956	1.00 (ref)^a,e^	1.20 (1.02–1.41)	1.43 (1.18–1.72)	1.19 (1.08–1.30)	0.0004	
Per category^c,e^		0.86 (0.79–0.94)	0.98 (0.92–1.05)	0.99 (0.90–1.09)			
P_trend_^c,e^		0.001	0.61	0.83			
**Fruits (g/day)**							0.69, 0.80
Low	7886	1.10 (0.93–1.30)	1.26 (1.09–1.46)	1.51 (1.27–1.80)	1.16 (1.07–1.26)	0.0004	
Medium	8030	1.13 (0.96–1.34)	1.25 (1.08–1.45)	1.46 (1.23–1.74)	1.14 (1.04–1.24)	0.003	
High	8033	1.00 (ref)	1.24 (1.07–1.44)	1.44 (1.21–1.72)	1.18 (1.08–1.29)	0.0002	
Per category		0.97 (0.89–1.06)	0.98 (0.92–1.05)	1.00 (0.90–1.10)			
P_trend_		0.48	0.52	0.92			
**Wine (g/day)**							0.029, 0.031
Non-consumers	6843	1.03 (0.86–1.22)	1.27 (1.09–1.49)	1.57 (1.32–1.88)	1.23 (1.14–1.34)	4 × 10^−7^	
Low	8472	0.93 (0.78–1.11)	1.14 (0.98–1.33)	1.29 (1.08–1.59)	1.16 (1.07–1.26)	0.0004	
High	8634	1.00 (ref)	1.01 (0.87–1.17)	1.17 (0.98–1.40)	1.08 (0.98–1.18)	0.11	
Per category		0.97 (0.88–1.08)	0.91 (0.85–0.98)	0.84 (0.75–0.93)			
P_trend_		0.61	0.01	0.001			
**Alcohol (g/day)**							0.73, 0.86
Abstainers	1448	1.19 (0.78–1.81)	1.63 (1.11–2.39)	2.26 (1.49–3.42)	1.34 (1.13–1.60)	0.001	
Low	17331	1.14 (0.81–1.60)	1.36 (0.97–1.91)	1.47 (1.04–2.08)	1.14 (1.07–1.20)	0.00002	
Medium	4150	1.18 (0.81–1.70)	1.06 (0.74–1.52)	1.64 (1.13–2.37)	1.15 (1.02–1.30)	0.03	
High	1020	1 (ref)	1.23 (0.82–1.85)	1.57 (0.99–2.50)	1.29 (1.02–1.63)	0.04	
Per category		0.98 (0.87–1.10)	0.85 (0.78–0.93)	0.96 (0.85–1.09)			
P_trend_		0.74	0.0004	0.57			

^a^Multivariable Cox proportional hazards model for incidence of cardiovascular disease (CVD) during follow-up with hazard ratio per each category of rs4977574 genotype and food or beverage intake assuming the highest category and AA genotype as reference.

^b^Multivariable Cox proportional hazards model for incidence of cardiovascular disease (CVD) during follow-up with hazard ratio per risk G allele using an additive genetic model.

^c^Multivariable Cox proportional hazards model for incidence of cardiovascular disease (CVD) during follow-up with hazard ratio per each higher food or beverage intake category with the lowest intake category as reference.

^d^Adjusted for age and sex.

^e^Adjusted for age, sex, BMI, SBP, season, method, total energy intake, leisure time physical activity, alcohol intake, smoking status, education, lipid lowering and antihypertensive treatment.

^f^Excluding BMI, SBP, antihypertensive and lipid lowering treatment from the adjustment model.

Concerning interaction with alcohol consumption, we observed that the HR for CVD incidence was lowest among AG genotype carriers in the medium alcohol intake group; however, this was not significantly different from the other intake groups as the interaction was not significant (Table 2). To determine if the lack of interaction between the total alcohol categories and rs4977574 on CVD risk was caused by an absence of statistical power, and as a sensitivity analysis, we excluded abstainers and performed an interaction analysis between tertiles of alcohol consumption and rs4977574 on CVD risk and observed that only among AG carriers the higher alcohol consumption associated with significantly lower incidence of CVD. However, no significant interaction was revealed (Additional file 1: Table S5).

**Figure 1:**
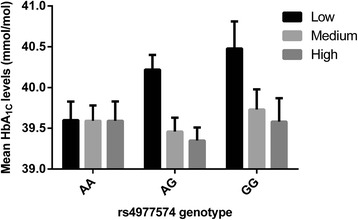
**Mean HbA**_**1C**_ **levels in tertiles of vegetable intake by rs4977574 genotype in MDC-CC.** The G allele was only associated with elevated HbA_1C_ levels among individuals in the lowest tertile of vegetable intake (β = 0.48 mmol/mol, SE = 0.18 per G allele, *P* = 0.009). Higher vegetable intake was only associated with lower HbA_1C_ among individuals with AG (β = −0.28 mmol/mol, SE = 0.12 per risk tertile, *P* = 0.019) and GG genotypes (β = −0.43 mmol/mol, SE = 0.20 per risk tertile, *P* = 0.032).

**Figure 2:**
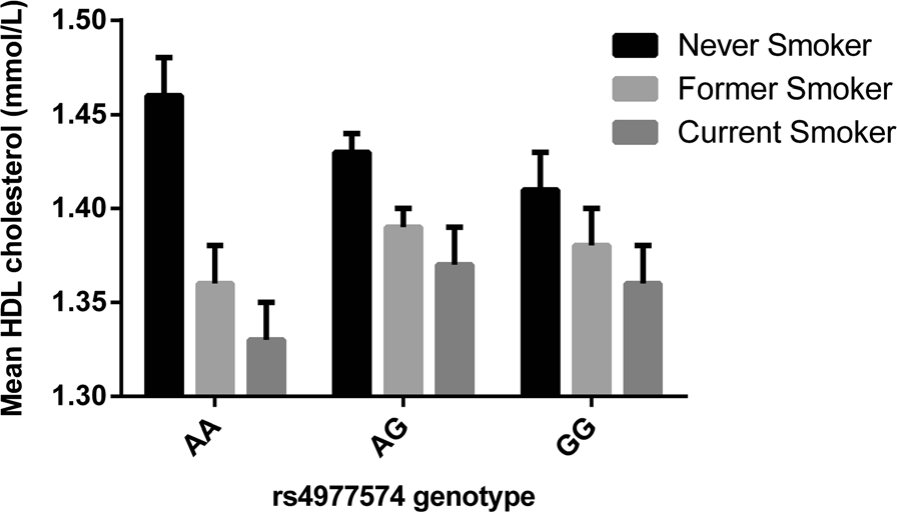
**Mean HDLC levels in smoking status categories by rs4977574 genotype in MDC-CC.** The G allele was only associated with lower levels of HDLC among never-smokers (β = −0.02 mmol/L, SE = 0.008 per G allele, *P* = 0.045). Higher risk categories of smoking were associated with lower HDLC for all genotypes. The magnitude of this association was largest among individuals with AA genotype (β = −0.05 mmol/L, SE = 0.01 per higher risk category, *P* = 1 × 10^−7^) compared with AG (β = −0.03 mmol/L, SE = 0.009 per higher risk category, *P* = 0.0004) and GG (β = −0.02 mmol/L, SE = 0.01 per higher risk category, P = 0.024) genotypes.

Consistent with earlier studies, rs4977574 did not associate with any of the known clinical or circulating risk factors of CVD (Table 1). However, stratification by environmental factors indicated that there was an interaction between rs4977574 and vegetable intake on baseline HbA_1C_ levels (P_interaction_ = 0.015), as well as smoking status on baseline HDLC levels (P_interaction_ = 0.049) (Additional file 1: Table S8). The rs4977574 G allele was only associated with elevated baseline HbA_1C_ levels in individuals within the lowest vegetable intake tertile (P_trend_ = 0.009), and with lower HDLC among never-smokers only (P_trend_ = 0.045). Moreover, higher vegetable intake was associated with lower HbA_1C_ levels among G allele carriers only (P_trend_ = 0.0002) (Figure 1). Smoking was strongly associated with lower HDLC levels in MDC-CC (P_trend_ = 1× 10^−10^), and the magnitude of this association was largest among individuals carrying no risk alleles (AA genotype, P_trend_ = 1 × 10^−7^) (Figure 2).

## Discussion

In this prospective study, we observed that dietary vegetable and wine intake modified the association of the chromosome 9p21 rs4977574 variant with CVD incidence. As expected, carriers of the rs4977574 G allele were associated with an increased incidence of CVD. Furthermore, a higher vegetable intake and moderate or high wine and alcohol consumption were associated with a lower incidence of CVD. However, the increased incidence of CVD among G allele carriers was restricted to individuals with a medium or high vegetable intake and to individuals reporting a zero or low consumption of wine. When stratified by rs4977574 genotype, the higher vegetable intake was only associated with a lower incidence of CVD in individuals with no risk alleles, while wine consumption was only associated with a lower incidence of CVD among risk allele carriers.

In line with earlier studies, the 9p21 variant did not associate with any of the traditional clinical risk factors in the present study. However, stratification by vegetable intake and smoking status suggested that it may associate with some of these risk factors, depending on the environment. We observed that presence of the G allele was associated with elevated HbA_1C_ levels among individuals in the lowest tertile of vegetable intake. Further, the G allele was only associated with lower HDLC levels among never-smokers. These nominally significant interactions indicate that the risk increase by 9p21 genetic variation may at least partially be mediated through deleterious effects on glucose and lipid metabolism, dependent on environmental risk factors of CVD.

Multiple interactions were observed between the rs4977574 variant and vegetable and wine intake with respect to CVD incidence. Concerning vegetable intake, the association with the genetic variant was attenuated and not significant among individuals at higher CVD risk because of low vegetable intake. This result is in line with our earlier observation that the associated effect of the 9p21 rs4977574 G allele was attenuated and not significant among smokers, i.e. among individuals already at higher risk because of an environmental risk factor for CVD [17]. Of further relevance to this are the results of an earlier study that reported an interaction between the 9p21 variant and a prudent diet score [16]. However, in contrast to our results, the associated effect of the risk allele was strongest among individuals in the lowest tertile of the prudent diet score. We also observed that wine intake was associated with a lower incidence of CVD, and that the risk increase caused by presence of the G allele was attenuated among consumers of wine. It therefore appears that the attenuation of the risk by the 9p21 variant may be mediated by both environmental risk factors (such as smoking and low vegetable intake) and protective factors (such as high wine intake) for CVD. Interaction analyses on CAD and stroke indicated similar tendencies with both vegetable and wine intake. However, due to decreased power in these subgroup analyses, statistical significance could not be attained. We did not find interaction with total alcohol intake although we observed the AG carriers to have lowest risk of CVD in the medium alcohol intake group and a strongly significantly decreased incidence of CVD by increased alcohol intake only among the AG genotype carriers.

It is important to recognize that vegetable and wine intakes are positively correlated and could reflect the same underlying interaction. However, when we mutually adjusted the analyses for these factors, the results remained similar. It is also of interest that the interaction between the 9p21 variant and wine intake on CVD risk was independent of total alcohol consumption, because an adjustment for total alcohol intake did not affect this result. Furthermore, adjusting for smoking status did not change the observed interactions between rs4977574 and vegetable or wine intake, indicating that these interactions are independent.

The mechanisms by which the 9p21 locus confers an increased risk of CVD remain incompletely understood. The SNPs in this locus are located in a 53-kb linkage disequilibrium (LD) block that lacks any protein-coding genes [2,4]. However, the large noncoding RNA antisense noncoding RNA in the INK locus (*ANRIL*) (also known as *CDKN2BAS*) has been mapped to the risk interval [27,28]. *ANRIL* expression has been shown to robustly associate with the 9p21 genotype and with the severity of atherosclerosis [29-32]. Although there is no conclusive evidence about the potential connection between *ANRIL* and CVD, several studies suggest a possible role in the epigenetic regulation of gene expression, and support *ANRIL* as the effector gene in 9p21 [33-36]. Interestingly, several of the bioactive compounds that have been recognized in nutritional epigenetics are found in wine and vegetables such as resveratrol (in red grapes), sulforaphane (in broccoli and sprouts), butyrate (a fermentation product of dietary fiber), and genistein (in fava beans and soya beans) [37-39]. If epigenetic mechanisms contribute to the association between 9p21 and CVD, our results suggest that they could potentially be influenced by nutritional or environmental factors and through gene–environment interactions.

It is noteworthy that an independent SNP in the *CDKN2A/B* locus near the 9p21 53-kb LD block has been robustly associated with type 2 diabetes [40,41]. This is particularly intriguing because we observed the rs4977574 risk allele to associate with elevated HbA_1C_ levels among individuals with a lower vegetable intake. Additionally, another interaction was observed between the 9p21 variant and HbA_1C_ on the risk of CAD in individuals with type 2 diabetes [42], such that the risk increase of CAD by the 9p21 risk variant was accentuated in individuals with elevated HbA_1C_ levels.

Our study has a number of limitations, which should be taken into account when analyzing results. First, baseline diet data were projected onto the entire follow-up period in the prospective analyses of CVD risk. Second, the inaccurate reporting of alcohol consumption is a known limitation in epidemiological studies, so could be a potential weakness [25]. Third, the cross-sectional analyses with CVD risk markers suffer from limited causal inference. Fourth, we did not correct the statistical analyses for multiple comparisons because the dietary variables are correlated. However, despite these limitations, the interactions observed between chromosome 9p21 variants and vegetable intake are supported by previous findings in the case/control INTERHEART study and the prospective FINRISK study [16]. Nevertheless, we should keep in mind that the observed significance levels of the interactions in our study were not very robust, so the possibility of false-positive findings cannot be excluded. On the other hand, our study has many important major strengths, including the high relative validity of our dietary assessment method, the combination of a diet diary with a questionnaire, the large sample size, the prospective design, the extensive follow-up of individuals through registers, and the comprehensive ascertainment and verification of CVD cases.

## Conclusions

Our results suggest that the chromosome 9p21 SNP rs4977574 interacts with several environmental risk factors to affect the incidence of CVD. Furthermore, the observed modifications of the association between rs4977574 and HbA_1C_ and HDLC levels by vegetable intake and smoking, respectively, provide evidence that the risk increase may include environmental interactions leading to derangements at the level of glucose and lipid metabolism.

## Additional file

Additional file 1:
**Table S1.** Characteristics of the Malmö Diet and Cancer Study population according to vegetable intake. **Table S2.** Characteristics of the Malmö Diet and Cancer Study population according to fruit intake. **Table S3.** Characteristics of the Malmö Diet and Cancer Study population according to wine consumption. **Table S4.** Characteristics of the Malmö Diet and Cancer Study population according to alcohol consumption. **Table S5.** Hazard ratio according to rs4977574 genotype for incident CVD in the Malmö Diet and Cancer study (n = 22,501) according to tertiles of alcohol consumption. **Table S6.** Hazard ratio for incident CAD in the Malmö Diet and Cancer Study (n = 23,949) according to rs4977574 genotype and dietary or alcohol intake categories. **Table S7.** Hazard ratio for incident stroke in the Malmö Diet and Cancer Study (n = 23,949) according to rs4977574 genotype and dietary or alcohol intake categories. **Table S8.** Interaction of rs4977574 variant with vegetable intake, wine intake, and smoking habits on CVD risk markers.

## Abbreviations

CVD: Cardiovascular disease
CAD: Coronary artery disease
MI: Myocardial infarction
MDCS: Malmö diet and cancer study
SNP: Single nucleotide polymorphism
MDC-CC: Malmö diet and cancer cardiovascular cohort
LDLC: Low-density lipoprotein cholesterol
HDLC: High-density lipoprotein cholesterol
TG: Triglycerides
hsCRP: High-sensitivity C-reactive protein
ICD: International classification of diseases
BMI: Body mass index
FPG: Fasting plasma glucose
HbA_1C_: Hemoglobin A_1C_
IFCC: International federation of clinical chemistry and laboratory medicine
GWAS: Genome-wide association study
SBP: Systolic blood pressure
DBP: Diastolic blood pressure
ANRIL: Antisense noncoding RNA in the INK locus
