# Supplementary material for: The chromosome 9p21 variant interacts with vegetable and wine intake to influence the risk of cardiovascular disease: a population based cohort study
Source: BMC Med Genet. 2014 Dec 31;15:1220. doi: 10.1186/s12881-014-0138-x (PMC4331503; doi:10.1186/s12881-014-0138-x)
Supplement: Additional file 1: — Table S1. Characteristics of the Malmö Diet and Cancer Study population according to vegetable intake. Table S2. Characteristics of the Malmö Diet and Cancer Study population according to fruit intake. Table S3. Characteristics of the Malmö Diet and Cancer Study population according to wine consumption. Table S4. Characteristics of the Malmö Diet and Cancer Study population according to alcohol consumption. Table S5. Hazard ratio according to rs4977574 genotype for incident CVD in the Malmö Diet and Cancer study (n = 22,501) according to tertiles of alcohol consumption. Table S6. Hazard ratio for incident CAD in the Malmö Diet and Cancer Study (n = 23,949) according to rs4977574 genotype and dietary or alcohol intake categories. Table S7. Hazard ratio for incident stroke in the Malmö Diet and Cancer Study (n = 23,949) according to rs4977574 genotype and dietary or alcohol intake categories. Table S8. Interaction of rs4977574 variant with vegetable intake, wine intake, and smoking habits on CVD risk markers. [file 12881_2014_138_MOESM1_ESM.docx]

**The chromosome 9p21 variant interacts with vegetable and wine intake to influence the risk of cardiovascular disease**

George Hindy^1^, Ulrika Ericson^1^, Viktor Hamrefors^2^, Isabel Drake^3^, Elisabet Wirfält^3^, Olle Melander^2^ and Marju Orho-Melander^1*^

^1^Diabetes and Cardiovascular Disease-Genetic Epidemiology; ^2^Hypertension and Cardiovascular Disease; and ^3^Nutrition Epidemiology, Department of Clinical Sciences in Malmö, Lund University, Sweden

*Corresponding author

Email addresses:

G. Hindy, [george.hindy@med.lu.se](mailto:george.hindy@med.lu.se)

U. Ericson, [ulrika.ericson@med.lu.se](mailto:ulrika.ericson@med.lu.se)

V. Hamrefors, [viktor.hamrefors@med.lu.se](mailto:viktor.hamrefors@med.lu.se)

I. Drake, [isabel.drake@med.lu.se](mailto:isabel.drake@med.lu.se)

E. Wirfält, [elisabet.wirfalt@med.lu.se](mailto:elisabet.wirfalt@med.lu.se)

O. Melander, [olle.melander@med.lu.se](mailto:olle.melander@med.lu.se)

M. Orho-Melander, [marju.orho-melander@med.lu.se](mailto:marju.orho-melander@med.lu.se)

**Supplementary Table 1 Characteristics of the Malmö Diet and Cancer Study population according to vegetable intake**

|  | Vegetable intake | | | HR (95%CI) ^a^ |  |
| --- | --- | --- | --- | --- | --- |
|  | Low | Medium | High | or β (SE) ^b^ | P*_trend_* |
| Total Number | 7952 | 8041 | 7956 |  |  |
| Sex (%women) | 57.0 | 63.5 | 66.6 |  |  |
| Incident CVD (%) | 1261 (15.9) | 1025 (12.7) | 878 (11.0) | 0.95 (0.91–0.996) | 0.032 |
| Age (years) | 59.1 ± 7.8 | 57.9 ± 7.7 | 56.6 ± 7.3 | −0.57 (0.06) | 2 ×10^−25^ |
| BMI (kg/m^2^) | 25.7 ± 4.0 | 25.6 ± 3.8 | 25.5 ± 3.9 | 0.16 (0.03) | 2 ×10^−07^ |
| Waist (cm) | 84.8 ± 15.8 | 83.2 ± 12.4 | 82.6 ± 15.1 | −0.20 (0.07) | 0.006 |
| SBP (mmHg) | 143 ± 20 | 141 ± 20 | 138 ± 19 | −0.42 (0.14) | 0.004 |
| DBP (mmHg) | 86 ± 10 | 85 ± 10 | 85 ± 10 | −0.21 (0.08) | 0.007 |
| FPG (mmol/L) ^c^ | 5.71 ± 0.89 | 5.61 ± 0.66 | 5.59 ± 0.82 | −0.04 (0.01) | 0.008 |
| HbA_1C_ (mmol/mol) ^c^ | 40.1 ± 5.13 | 39.6 ± 4.47 | 39.5 ± 5.21 | −0.18 (0.09) | 0.03 |
| LDLC (mmol/L) ^c^ | 4.19 ± 0.96 | 4.16 ± 0.96 | 4.18 ± 1.02 | 0.01 (0.02) | 0.87 |
| HDLC (mmol/L) ^c^ | 1.36 ± 0.37 | 1.40 ± 0.37 | 1.42 ± 0.38 | 0.02 (0.006) | 0.01 |
| Triglycerides (mmol/L) ^c^ | 1.40 ± 0.77 | 1.33 ± 0.71 | 1.30 ± 0.78 | −0.03 (0.01) | 0.006 |
| hsCRP ^c^ | 0.27 ± 0.43 | 0.25 ± 0.43 | 0.24 ± 0.41 | −0.009 (0.008) | 0.03 |
| Total energy intake (kcal/day) | 2208 ± 648 | 2256 ± 625 | 2365 ± 675 | 95 (4.6) | 2×10^−93^ |
| Vegetables (g/day) | 90 ± 28 | 164 ± 21 | 289 ± 90 | 98 (0.5) | 0 |
| Fruits (g/day) | 153 ± 109 | 189 ± 115 | 242 ± 136 | 38 (1) | 0 |
| Wine (g/) | 35.2 ± 56.6 | 42.9 ± 59.3 | 48.6 ± 63.3 | 2.8 (0.4) | 3 ×10^−13^ |
| Alcohol (g/day) | 10.2 ± 13.0 | 10.8 ± 12.1 | 11.4 ± 12.6 | 0.35 (0.10) | 0.0002 |

Data represented as mean ± standard deviation

^a^ Multivariable Cox proportional hazards model for incidence of cardiovascular disease (CVD) during follow-up adjusting for age, sex, BMI, SBP, season, method, total energy intake, leisure time physical activity, alcohol intake, smoking status, education, lipid lowering and antihypertensive treatment with HR referring to hazard ratio per tertile of vegetable intake with the lowest tertile as reference

^b^ Linear regression analyses of tertiles of vegetable intake with quantitative traits or characteristics at baseline adjusting for age, sex, BMI, SBP, season, method, total energy intake, leisure time physical activity, alcohol intake, smoking status, education, lipid lowering and antihypertensive treatment when appropriate with β referring to associated effect estimate per tertile of vegetable intake with the lowest tertile as reference

^c^ In MDC-CC only, N=4,828 (AA N=1,460; AG N=2,381; GG N=987)

CI, Confidence Interval; CVD, cardiovascular disease; SE, standard error; BMI, Body Mass Index; SBP, Systolic Blood Pressure; DBP, Diastolic Blood Pressure; FPG, Fasting Plasma Glucose; HbA_1C_, Hemoglobin A_1C_; LDLC, Low-Density Lipoprotein Cholesterol; HDLC, High-Density Lipoprotein Cholesterol; hsCRP, High-Sensitivity C-Reactive Protein

**Supplementary Table 2 Characteristics of the Malmö Diet and Cancer Study population according to fruit intake**

|  | Fruit intake | | | HR (95%CI) ^a^ |  |
| --- | --- | --- | --- | --- | --- |
|  | Low | Medium | High | or β (SE) ^b^ | P*_trend_* |
| Total Number | 7886 | 8030 | 8033 |  |  |
| Sex (%women) | 51.6 | 64.7 | 70.6 |  |  |
| Incident CVD (%) | 1126 (14.3) | 1069 (13.3) | 969 (12.1) | 0.98 (0.93–1.02) | 0.32 |
| Age (years) | 57.2 ± 7.6 | 58.3 ± 7.8 | 58.2 ± 7.6 | 0.61 (0.06) | 2×10^−27^ |
| BMI (kg/m^2^) | 25.4 ± 3.9 | 25.6 ± 3.9 | 25.8 ± 4.0 | 0.33 (0.03) | 6×10^−26^ |
| Waist (cm) | 84.9 ± 13.0 | 83.3 ± 17.5 | 82.4 ± 12.4 | −0.32 (0.07) | 0.00002 |
| SBP (mmHg) | 141 ± 20 | 141 ± 20 | 140 ± 20 | −0.54 (0.15) | 0.0003 |
| DBP (mmHg) | 86 ± 10 | 85 ± 10 | 85 ± 10 | −0.19 (0.08) | 0.01 |
| FPG (mmol/L) ^c^ | 5.72 ± 0.90 | 5.60 ± 0.69 | 5.59 ± 0.79 | −0.04 (0.01) | 0.01 |
| HbA_1C_ (mmol/mol) ^c^ | 40.2 ± 5.40 | 39.4 ± 4.54 | 39.5 ± 4.95 | −0.36 (0.09) | 0.00006 |
| LDLC (mmol/L) ^c^ | 4.16 ± 0.98 | 4.17 ± 0.96 | 4.20 ± 1.01 | 0.002 (0.018) | 0.92 |
| HDLC (mmol/L) ^c^ | 1.35 ± 0.35 | 1.41 ± 0.39 | 1.41 ± 0.37 | −0.001 (0.006) | 0.78 |
| Triglycerides (mmol/L) ^c^ | 1.40 ± 0.84 | 1.31 ± 0.72 | 1.31 ± 0.71 | −0.01 (0.01) | 0.28 |
| hsCRP ^c^ | 0.28 ± 0.44 | 0.23 ± 0.36 | 0.25 ± 0.46 | −0.004 (0.008) | 0.05 |
| Total energy intake (kcal/day) | 2249 ± 662 | 2245 ± 633 | 2334 ± 660 | 118 (5) | 2×10^−141^ |
| Vegetables (g/day) | 146 ± 79 | 177 ± 89 | 219 ± 113 | 33 (0.8) | 0 |
| Fruits (g/day) | 76 ± 35 | 173 ± 29 | 334 ± 107 | 126 (0.6) | 0 |
| Wine (g/) | 45.6 ± 66.9 | 42.0 ± 57.6 | 39.2 ± 55.0 | 1.18 (0.39) | 0.003 |
| Alcohol (g/day) | 13.1 ± 14.9 | 10.2 ± 11.7 | 9.0 ± 10.4 | −1.49 (0.10) | 2×10^−53^ |

Data represented as mean ± standard deviation

^a^ Multivariable Cox proportional hazards model for incidence of cardiovascular disease (CVD) during follow-up adjusting for age, sex, BMI, SBP, season, method, total energy intake, leisure time physical activity, alcohol intake, smoking status, education, lipid lowering and antihypertensive treatment with HR referring to hazard ratio per tertile of fruit intake with the lowest tertile as reference

^b^ Linear regression analyses of tertiles of vegetable intake with quantitative traits or characteristics at baseline adjusting for age, sex, BMI, SBP, season, method, total energy intake, leisure time physical activity, alcohol intake, smoking status, education, lipid lowering and antihypertensive treatment when appropriate with β referring to associated effect estimate per tertile of fruit intake with the lowest tertile as reference

^c^ In MDC-CC only, N=4,828 (AA N=1,460; AG N=2,381; GG N=987)

CI, Confidence Interval; CVD, cardiovascular disease; SE, standard error; BMI, Body Mass Index; SBP, Systolic Blood Pressure; DBP, Diastolic Blood Pressure; FPG, Fasting Plasma Glucose; HbA_1C_, Hemoglobin A_1C_; LDLC, Low-Density Lipoprotein Cholesterol; HDLC, High-Density Lipoprotein Cholesterol; hsCRP, High-Sensitivity C-Reactive Protein

**Supplementary Table 3 Characteristics of the Malmö Diet and Cancer Study population according to wine consumption**

|  | Wine intake | | | HR (95%CI) ^a^ |  |
| --- | --- | --- | --- | --- | --- |
|  | Non-consumers | Low | High | or β (SE) ^b^ | P*_trend_* |
| Total Number | 6843 | 8472 | 8634 |  |  |
| Sex (%women) | 58.6 | 66.3 | 61.5 |  |  |
| Incident CVD (%) | 1156 (16.9) | 1088 (12.8) | 920 (10.7) | 0.91 (0.86–0.96) | 0.0003 |
| Age (years) | 59.3 ± 7.6 | 58.3 ± 7.7 | 56.3 ± 7.4 | −0.51 (0.06) | 8×10^−16^ |
| BMI (kg/m^2^) | 26.1 ± 4.3 | 25.6 ± 3.9 | 25.2 ± 3.5 | −0.30 (0.04) | 3×10^−17^ |
| Waist (cm) | 85.6 ± 18.6 | 82.8 ± 12.5 | 82.5 ± 12.4 | −0.39 (0.08) | 3×10^−6^ |
| SBP (mmHg) | 143 ± 20 | 141 ± 20 | 139 ± 20 | −0.25 (0.17) | 0.14 |
| DBP (mmHg) | 86 ± 10 | 85 ± 10 | 85 ± 10 | 0.11 (0.09) | 0.20 |
| FPG (mmol/L) ^c^ | 5.69 ± 0.86 | 5.58 ± 0.74 | 5.63 ± 0.70 | −0.03 (0.02) | 0.08 |
| HbA_1C_ (mmol/mol) ^c^ | 40.5 ± 5.81 | 39.6 ± 4.73 | 39.1 ± 4.34 | −0.35 (0.10) | 0.0005 |
| LDLC (mmol/L) ^c^ | 4.21 ± 1.02 | 4.19 ± 0.97 | 4.14 ± 0.97 | 0.02 (0.02) | 0.32 |
| HDLC (mmol/L) ^c^ | 1.30 ± 0.34 | 1.41 ± 0.37 | 1.46 ± 0.39 | 0.05 (0.007) | 2×10^−15^ |
| Triglycerides (mmol/L) ^c^ | 1.41 ± 0.75 | 1.31 ± 0.69 | 1.30 ± 0.82 | −0.05 (0.02) | 0.0004 |
| hsCRP ^c^ | 0.29 ± 0.47 | 0.23 ± 0.41 | 0.24 ± 0.40 | −0.01 (0.009) | 0.05 |
| Total energy intake (kcal/day) | 2284 ± 720 | 2239 ± 624 | 2306 ± 622 | −42.9 (5.42) | 3×10^-15^ |
| Vegetables (g/day) | 164 ± 99 | 181 ± 98 | 193 ± 100 | 9.16 (0.91) | 1×10^−23^ |
| Fruits (g/day) | 193 ± 133 | 201 ± 124 | 190 ± 122 | 3.62 (1.14) | 0.001 |
| Wine (g) | 0 | 18.1 ± 12.0 | 99 ± 68 | 36.6 (0.38) | 0 |
| Alcohol (g/day) | 3.95 ± 8.85 | 7.24 ± 8.44 | 19.6 ± 13.4 | 7.90 (0.08) | 0 |

Data represented as mean ± standard deviation

^a^ Multivariable Cox proportional hazards model for incidence of cardiovascular disease (CVD) during follow-up adjusting for age, sex, BMI, SBP, season, method, total energy intake, leisure time physical activity, alcohol intake, smoking status, education, lipid lowering and antihypertensive treatment with HR referring to hazard ratio per wine intake category with non-consumers as reference

^b^ Linear regression analyses of tertiles of vegetable intake with quantitative traits or characteristics at baseline adjusting for age, sex, BMI, SBP, season, method, total energy intake, leisure time physical activity, alcohol intake, smoking status, education, lipid lowering and antihypertensive treatment when appropriate with β referring to associated effect estimate per wine intake category with non-consumers as reference

^c^ In MDC-CC only, N=4,828 (AA N=1,460; AG N=2,381; GG N=987)

CI, Confidence Interval; CVD, cardiovascular disease; SE, standard error; BMI, Body Mass Index; SBP, Systolic Blood Pressure; DBP, Diastolic Blood Pressure; FPG, Fasting Plasma Glucose; HbA_1C_, Hemoglobin A_1C_; LDLC, Low-Density Lipoprotein Cholesterol; HDLC, High-Density Lipoprotein Cholesterol; hsCRP, High-Sensitivity C-Reactive Protein

**Supplementary Table 4 Characteristics of the Malmö Diet and Cancer Study population according to alcohol consumption**

|  | Alcohol intake | | | | HR (95%CI) ^a^ |  |
| --- | --- | --- | --- | --- | --- | --- |
|  | Abstainers | Low | Medium | High | or β (SE) ^b^ | P*_trend_* |
| Total Number | 1448 | 17331 | 4150 | 1020 |  |  |
| Sex (%women) | 74.1 | 65.7 | 51.1 | 34.9 |  |  |
| Incident CVD (%) | 241 (16.6) | 2277 (13.1) | 508 (12.2) | 138 (13.5) | 0.92 (0.86–0.98) | 0.006 |
| Age (years) | 60.3 ± 7.5 | 58.2 ± 7.7 | 56.4 ± 7.3 | 55.9 ± 6.8 | −1.10 (0.07) | 3×10^−49^ |
| BMI (kg/m^2^) | 26.4 ± 4.7 | 25.6 ± 3.9 | 25.4 ± 3.6 | 25.8 ± 3.7 | −0.04 (0.04) | 0.37 |
| Waist (cm) | 83.9 ± 13.0 | 82.9 ± 15.0 | 84.6 ± 13.0 | 89.0 ± 13.2 | 0.15 (0.10) | 0.13 |
| SBP (mmHg) | 144 ± 20 | 141 ± 20 | 140 ± 20 | 142 ± 19 | 1.21 (0.20) | 6×10^−10^ |
| DBP (mmHg) | 86 ± 10 | 85 ± 10 | 86 ± 10 | 87 ± 10 | 0.76 (0.10) | 1×10^-13^ |
| FPG (mmol/L) ^c^ | 5.62 ± 0.70 | 5.60 ± 0.79 | 5.68 ± 0.75 | 6.04 ± 1.12 | 0.09 (0.02) | 6×10^−6^ |
| HbA_1C_ (mmol/mol) ^c^ | 40.9 ± 4.56 | 39.7 ± 5.06 | 39.2 ± 4.42 | 39.6 ± 5.41 | −0.31 (0.13) | 0.02 |
| LDLC (mmol/L) ^c^ | 4.36 ± 1.28 | 4.18 ± 1.00 | 4.12 ± 0.93 | 4.12 ± 0.92 | −0.01 (0.03) | 0.82 |
| HDLC (mmol/L) ^c^ | 1.27 ± 0.31 | 1.40 ± 0.37 | 1.42 ± 0.38 | 1.41 ± 0.39 | 0.10 (0.009) | 2×10^−31^ |
| Triglycerides (mmol/L) ^c^ | 1.48 ± 0.73 | 1.31 ± 0.68 | 1.34 ± 0.80 | 1.70 ± 1.55 | 0.03 (0.02) | 0.54 |
| hsCRP ^b^ | 0.31 ± 0.49 | 0.24 ± 0.43 | 0.24 ± 0.38 | 0.29 ± 0.51 | −0.003 (0.01) | 0.58 |
| Total energy intake (kcal/day) | 2131 ± 747 | 2228 ± 630 | 2416 ± 623 | 2721 ± 740 | 96.7 (6.32) | 1×10^-52^ |
| Vegetables (g/day) | 161 ± 105 | 180 ± 99 | 188 ± 95 | 188 ± 111 | 3.32 (1.07) | 0.002 |
| Fruits (g/day) | 208 ± 142 | 201 ± 125 | 174 ± 118 | 149 ± 119 | −20.4 (1.33) | 9×10^−53^ |
| Wine (g) | 0 ± 0 | 25.9 ± 31.8 | 94.9 ± 62.9 | 167 ± 133 | 59.4 (0.52) | 0 |
| Alcohol (g/day) | 0 ± 0 | 6.20 ± 5.17 | 23.8 ± 6.03 | 50.8 ± 17.8 | 16.4 (0.08) | 0 |

Data represented as mean ± standard deviation

^a^ Multivariable Cox proportional hazards model for incidence of cardiovascular disease (CVD) during follow-up adjusting for age, sex, BMI, SBP, season, method, total energy intake, leisure time physical activity, alcohol intake, smoking status, education, lipid lowering and antihypertensive treatment with HR referring to hazard ratio per alcohol intake category with abstainers as reference

^b^ Linear regression analyses of tertiles of vegetable intake with quantitative traits or characteristics at baseline adjusting for age, sex, BMI, SBP, season, method, total energy intake, leisure time physical activity, alcohol intake, smoking status, education, lipid lowering and antihypertensive treatment when appropriate with β referring to associated effect estimate per alcohol intake category with abstainers as reference

^c^ In MDC-CC only, N=4,828 (AA N=1,460; AG N=2,381; GG N=987)

CI, Confidence Interval; CVD, cardiovascular disease; SE, standard error; BMI, Body Mass Index; SBP, Systolic Blood Pressure; DBP, Diastolic Blood Pressure; FPG, Fasting Plasma Glucose; HbA_1C_, Hemoglobin A_1C_; LDLC, Low-Density Lipoprotein Cholesterol; HDLC, High-Density Lipoprotein Cholesterol; hsCRP, High-Sensitivity C-Reactive Protein

**Supplementary Table 5 Hazard ratio according to rs4977574 genotype for incident CVD in the Malmö Diet and Cancer study (n = 22,501) according to tertiles of alcohol consumption**

|  |  | Hazard Ratio (95% Confidence Interval) | | | | P*_trend_* ^b, d^ | P*_interaction_* ^e, f^ |
| --- | --- | --- | --- | --- | --- | --- | --- |
|  |  | AA | AG | GG | Additive Model ^b, d^ |  |  |
|  | *N* | 6853 | 11111 | 4537 |  |  |  |
| **Alcohol (g/day)** |  |  |  |  |  |  | 0.24, 0.26 |
| Low | 7469 | 1.01 (0.85–1.19) | 1.25 (1.08–1.46) | 1.45 (1.22–1.73) | 1.18 (1.09–1.29) | 0.0001 |  |
| Medium | 7513 | 0.91 (0.77–1.09) | 1.16 (1.00–1.35) | 1.19 (1.00–1.43) | 1.16 (1.06–1.27) | 0.002 |  |
| High | 7519 | 1 (ref) | 0.96 (0.83–1.12) | 1.26 (1.07–1.50) | 1.10 (1.01–1.20) | 0.03 |  |
| Per category ^c, e^ |  | 1.00 (0.92–1.10) | 0.87 (0.82–0.93) | 0.94 (0.85–1.03) |  |  |  |
| P*_trend_* ^c, e^ |  | 0.97 | 0.00009 | 0.19 |  |  |  |

^a^ Multivariable Cox proportional hazards model for incidence of cardiovascular disease (CVD) during follow-up with hazard ratio per each category of rs4977574 genotype and alcohol intake assuming the highest category and AA genotype as reference

^b^ Multivariable Cox proportional hazards model for incidence of cardiovascular disease (CVD) during follow-up with hazard ratio per risk G allele using an additive genetic model

^c^ Multivariable Cox proportional hazards model for incidence of cardiovascular disease (CVD) during follow-up with hazard ratio per each tertile of alcohol intake with the lowest tertile as reference.

^d^ Adjusted for age and sex

^e^ Adjusted for age, sex, BMI, SBP, season, method, total energy intake, leisure time physical activity, alcohol intake, smoking status, education, lipid lowering and antihypertensive treatment

^f^ Excluding BMI, SBP, antihypertensive and lipid lowering treatment from the adjustment model

**Supplementary Table 6 Hazard ratio for incident CAD in the Malmö Diet and Cancer Study (n = 23,949) according to rs4977574 genotype and dietary or alcohol intake categories**

|  |  | HR (95% CI) | | | | P*_trend_* ^b, d^ | P*_interaction_* ^e, f^ |
| --- | --- | --- | --- | --- | --- | --- | --- |
|  |  | AA | AG | GG | Additive Model ^b, d^ |  |  |
|  | *n* | 7325 | 11777 | 4847 |  |  |  |
| **Vegetables (g/day)** |  |  |  |  |  |  | 0.20, 0.25 |
| Low | 7952 | 1.19 (0.96–1.48) | 1.16 (0.95–1.42) | 1.46 (1.16–1.83) | 1.09 (0.98–1.20) | 0.11 |  |
| Medium | 8041 | 0.79 (0.62–1.00) | 1.20 (0.98–1.46) | 1.31 (1.03–1.66) | 1.29 (1.15–1.44) | 0.00002 |  |
| High | 7956 | 1.00 (ref)^a, e^ | 1.12 (0.91–1.38) | 1.44 (1.13–1.83) | 1.19 (1.05–1.35) | 0.005 |  |
| Per category ^c, e^ |  | 0.90 (0.90–1.02) | 0.98 (0.90–1.06) | 0.99 (0.88–1.12) |  |  |  |
| P*_trend_* ^c, e^ |  | 0.09 | 0.58 | 0.84 |  |  |  |
| **Fruits (g/day)** |  |  |  |  |  |  | 1.00, 0.87 |
| Low | 7886 | 1.04 (0.84–1.30) | 1.30 (1.07–1.58) | 1.59 (1.27–1.99) | 1.23 (1.11–1.37) | 0.0001 |  |
| Medium | 8030 | 1.14 (0.92–1.42) | 1.29 (1.06–1.56) | 1.33 (1.05–1.68) | 1.08 (0.97–1.21) | 0.16 |  |
| High | 8033 | 1.00 (ref) | 1.10 (0.89–1.34) | 1.55 (1.24–1.95) | 1.23 (1.09–1.38) | 0.0002 |  |
| Per category |  | 1.02 (0.91–1.14) | 0.92 (0.84–1.00) | 0.97 (0.86–1.10) |  |  |  |
| P*_trend_* |  | 0.76 | 0.044 | 0.63 |  |  |  |
| **Wine (g/day)** |  |  |  |  |  |  | 0.13, 0.14 |
| Non-consumers | 6843 | 1.16 (0.92–1.47) | 1.50 (1.22–1.85) | 1.87 (1.49–2.37) | 1.27 (1.14–1.40) | 7 ×10^−6^ |  |
| Low | 8472 | 1.10 (0.88–1.39) | 1.26 (1.02–1.55) | 1.44 (1.13–1.83) | 1.13 (1.01–1.26) | 0.03 |  |
| High | 8634 | 1.00 (ref) | 1.03 (0.84–1.27) | 1.28 (1.01–1.63) | 1.13 (1.00–1.28) | 0.05 |  |
| Per category |  | 0.96 (0.84–1.09) | 0.83 (0.76–0.92) | 0.81 (0.70–0.93) |  |  |  |
| P*_trend_* |  | 0.50 | 0.0002 | 0.002 |  |  |  |
| **Alcohol (g/day)** |  |  |  |  |  |  | 0.92, 0.78 |
| Abstainers | 1448 | 1.38 (0.82–2.34) | 1.32 (0.80–2.18) | 2.34 (1.38–3.96) | 1.26 (1.00–1.60) | 0.06 |  |
| Low | 17331 | 1.10 (0.71–1.69) | 1.35 (0.88–2.07) | 1.48 (0.95–2.28) | 1.16 (1.08–1.25) | 0.0001 |  |
| Medium | 4150 | 1.06 (0.66–1.70) | 1.06 (0.68–1.67) | 1.58 (0.98–2.52) | 1.20 (1.02–1.40) | 0.03 |  |
| High | 1020 | 1 (ref) | 1.04 (0.62–177) | 1.52 (0.85–2.73) | 1.28 (0.95–1.74) | 0.11 |  |
| Per category |  | 0.92 (0.79–1.07) | 0.86 (0.77–0.96) | 0.94 (0.80–1.10) |  |  |  |
| P*_trend_* |  | 0.29 | 0.01 | 0.45 |  |  |  |

^a^ Multivariable Cox proportional hazards model for incidence of coronary artery disease (CAD) during follow-up with hazard ratio per each category of rs4977574 genotype and food or beverage intake assuming the highest category and AA genotype as reference

^b^ Multivariable Cox proportional hazards model for incidence of coronary artery disease (CAD) during follow-up with hazard ratio per risk G allele using an additive genetic model

^c^ Multivariable Cox proportional hazards model for incidence of coronary artery disease (CAD) during follow-up with hazard ratio per each higher food or beverage intake category with the lowest intake category as reference.

^d^ Adjusted for age and sex

^e^ Adjusted for age, sex, BMI, SBP, season, method, total energy intake, leisure time physical activity, alcohol intake, smoking status, education, lipid lowering and antihypertensive treatment

^f^ Excluding BMI, SBP, antihypertensive and lipid lowering treatment from the adjustment model

**Supplementary Table 7 Hazard ratio for incident stroke in the Malmö Diet and Cancer Study (n = 23,949) according to rs4977574 genotype and dietary or alcohol intake categories**

|  |  | OR (95% CI) | | | | P*_trend_* ^b, d^ | P*_interaction_* ^e, f^ |
| --- | --- | --- | --- | --- | --- | --- | --- |
|  |  | AA | AG | GG | Additive Model ^b, d^ |  |  |
|  | *n* | 7325 | 11777 | 4847 |  |  |  |
| **Vegetables (g/day)** |  |  |  |  |  |  | 0.051, 0.07 |
| Low | 7952 | 1.47 (1.15–1.88) | 1.23 (0.98–1.56) | 1.55 (1.19–2.02) | 1.02 (0.91–1.14) | 0.78 |  |
| Medium | 8041 | 1.02 (0.79–1.33) | 1.31 (1.04–1.65) | 1.48 (1.13–1.94) | 1.22 (1.08–1.38) | 0.002 |  |
| High | 7956 | 1.00 (ref)^a, e^ | 1.29 (1.02–1.65) | 1.40 (1.06–1.87) | 1.16 (1.02–1.33) | 0.03 |  |
| Per category ^c, e^ |  | 0.82 (0.72–0.93) | 1.00 (0.91–1.09) | 0.97 (0.85–1.12) |  |  |  |
| P*_trend_* ^c, e^ |  | 0.002 | 0.97 | 0.70 |  |  |  |
| **Fruits (g/day)** |  |  |  |  |  |  | 0.14, 0.23 |
| Low | 7886 | 1.36 (1.07–1.73) | 1.21 (0.97–1.52) | 1.48 (1.14–1.93) | 1.05 (0.93–1.19) | 0.42 |  |
| Medium | 8030 | 1.20 (0.94–1.53) | 1.22 (0.98–1.53) | 1.61 (1.25–2.07) | 1.16 (1.03–1.31) | 0.02 |  |
| High | 8033 | 1.00 (ref) | 1.42 (1.14–1.76) | 1.37 (1.05–1.78) | 1.15 (1.01–1.30) | 0.03 |  |
| Per category |  | 0.88 (0.78–0.99) | 1.08 (0.98–1.18) | 1.02 (0.89–1.17) |  |  |  |
| P*_trend_* |  | 0.04 | 0.13 | 0.82 |  |  |  |
| **Wine (g/day)** |  |  |  |  |  |  | 0.20, 0.19 |
| Non-consumers | 6843 | 0.88 (0.69–1.13) | 0.95 (0.76–1.19) | 1.14 (0.88–1.48) | 1.16 (1.03–1.31) | 0.01 |  |
| Low | 8472 | 0.75 (0.59–0.97) | 0.95 (0.77–1.18) | 1.10 (0.86–1.42) | 1.20 (1.06–1.35) | 0.003 |  |
| High | 8634 | 1.00 (ref) | 0.92 (0.74–1.13) | 1.02 (0.78–1.32) | 1.00 (0.88–1.13) | 0.94 |  |
| Per category |  | 1.02 (0.88–1.18) | 1.06 (0.95–1.18) | 0.88 (0.75–1.02) |  |  |  |
| P*_trend_* |  | 0.79 | 0.34 | 0.09 |  |  |  |
| **Alcohol (g/day)** |  |  |  |  |  |  | 0.43, 0.51 |
| Abstainers | 1448 | 0.86 (0.47–1.57) | 1.74 (1.03–2.93) | 1.70 (0.95–3.07) | 1.36 (1.07–1.73) | 0.01 |  |
| Low | 17331 | 1.02 (0.63–1.64) | 1.14 (0.71–1.82) | 1.22 (0.76–1.98) | 1.09 (1.00–1.18) | 0.04 |  |
| Medium | 4150 | 1.14 (0.68–1.90) | 0.83 (0.50–1.38) | 1.50 (0.89–2.53) | 1.11 (0.93–1.33) | 0.24 |  |
| High | 1020 | 1 (ref) | 1.24 (0.70–2.20) | 1.28 (0.64–2.54) | 1.14 (0.81–1.60) | 0.45 |  |
| Per category |  | 1.08 (0.92–1.27) | 0.81 (0.71–0.93) | 0.99 (0.83–1.19) |  |  |  |
| P*_trend_* |  | 0.74 | 0.002 | 0.94 |  |  |  |

^a^ Multivariable Cox proportional hazards model for incidence of stroke during follow-up with hazard ratio per each category of rs4977574 genotype and food or beverage intake assuming the highest category and AA genotype as reference

^b^ Multivariable Cox proportional hazards model for incidence of stroke during follow-up with hazard ratio per risk G allele using an additive genetic model

^c^ Multivariable Cox proportional hazards model for incidence of stroke during follow-up with hazard ratio per each higher food or beverage intake category with the lowest intake category as reference.

^d^ Adjusted for age and sex

^e^ Adjusted for age, sex, BMI, SBP, season, method, total energy intake, leisure time physical activity, alcohol intake, smoking status, education, lipid lowering and antihypertensive treatment

^f^ Excluding BMI, SBP, antihypertensive and lipid lowering treatment from the adjustment model

**Supplementary Table 8 Interaction of rs4977574 variant with vegetable intake, wine intake, and smoking habits on CVD risk markers**

|  |  | Vegetable × 9p21 | |  | Wine × 9p21 | |  | Smoking × 9p21 | |
| --- | --- | --- | --- | --- | --- | --- | --- | --- | --- |
|  |  | P*_interaction_* ^a^ | P*_interaction_* ^c^ |  | P*_interaction_* ^a^ | P*_interaction_* ^c^ |  | P*_interaction_*^b^ | P*_interaction_* ^c^ |
| SBP (mmHg) |  | 0.52 | 0.41 |  | 0.70 | 0.78 |  | 0.94 | 0.74 |
| DBP (mmHg) |  | 0.93 | 0.75 |  | 0.78 | 0.70 |  | 0.79 | 0.55 |
| FPG (mmol/L) |  | 0.63 | 0.55 |  | 0.19 | 0.31 |  | 0.88 | 0.95 |
| HbA_1C_ (mmol/mol) |  | 0.015 | 0.015 |  | 0.53 | 0.63 |  | 0.85 | 0.96 |
| LDLC (mmol/L) |  | 0.65 | 0.60 |  | 0.75 | 0.77 |  | 0.53 | 0.62 |
| HDLC (mmol/L) |  | 0.54 | 0.45 |  | 0.24 | 0.40 |  | 0.049 | 0.024 |
| Triglycerides |  | 0.13 | 0.10 |  | 0.55 | 0.72 |  | 0.15 | 0.095 |
| hsCRP |  | 0.57 | 0.49 |  | 0.41 | 0.30 |  | 0.40 | 0.29 |

^a^ Adjusted for age, sex, BMI, SBP, season, method, total energy intake, physical activity leisure time, smoking status, education, lipid lowering and antihypertensive treatment

^b^ Adjusted for age, sex, BMI, SBP, total energy intake, physical activity leisure time, education, lipid lowering and antihypertensive treatment

^c^ Excluding BMI, SBP, antihypertensive and lipid-lowering treatments from the adjustment model when needed

SBP, Systolic Blood Pressure; DBP, Diastolic Blood Pressure; FPG, Fasting Plasma Glucose; HbA_1C_, Hemoglobin A_1C_; LDLC, Low-Density Lipoprotein Cholesterol; HDLC, High-Density Lipoprotein Cholesterol; hsCRP, High-Sensitivity C-Reactive Protein
